# Supplementary figures and images for: Evaluation of the External RNA Controls Consortium (ERCC) reference material using a modified Latin square design
Source: BMC Biotechnol. 2016 Jun 24;16:54. doi: 10.1186/s12896-016-0281-x (PMC4921035; doi:10.1186/s12896-016-0281-x)

Log2 Ratio

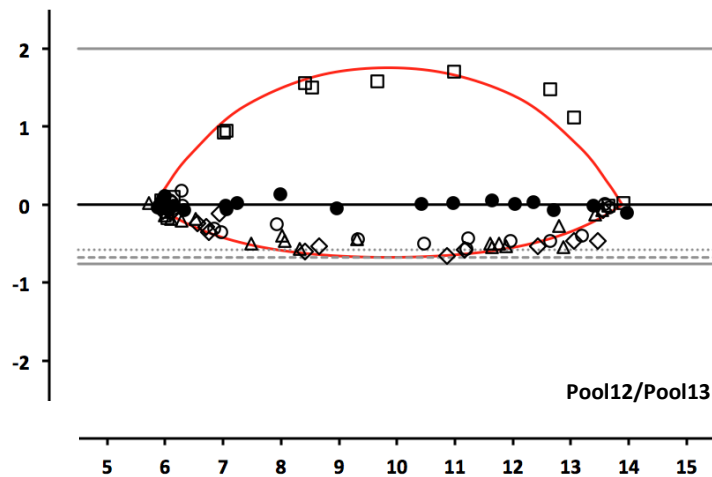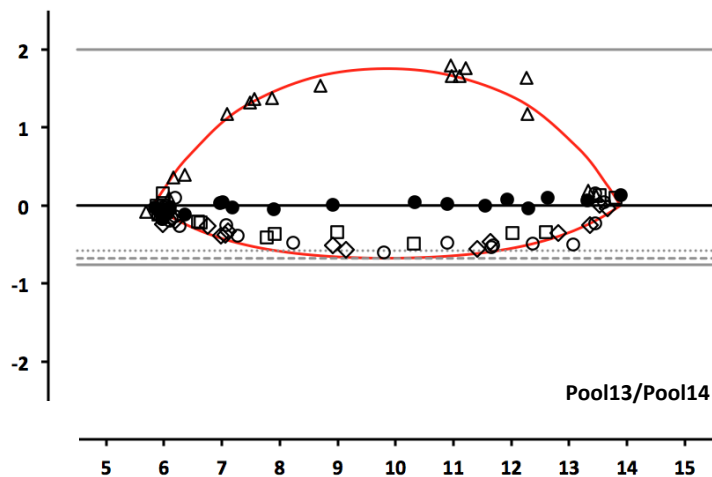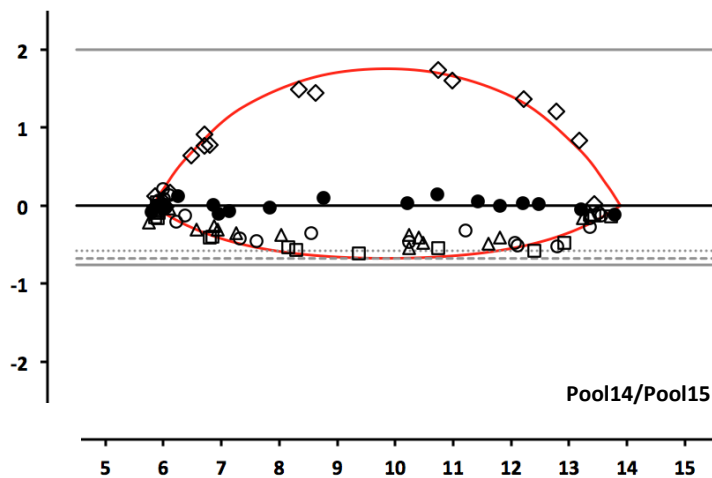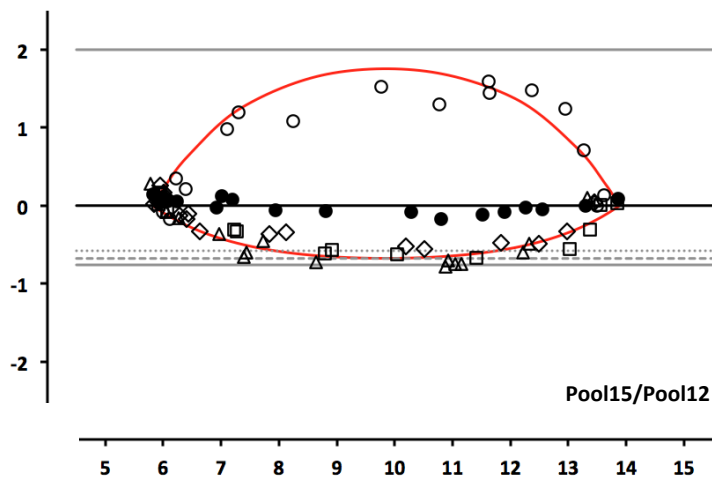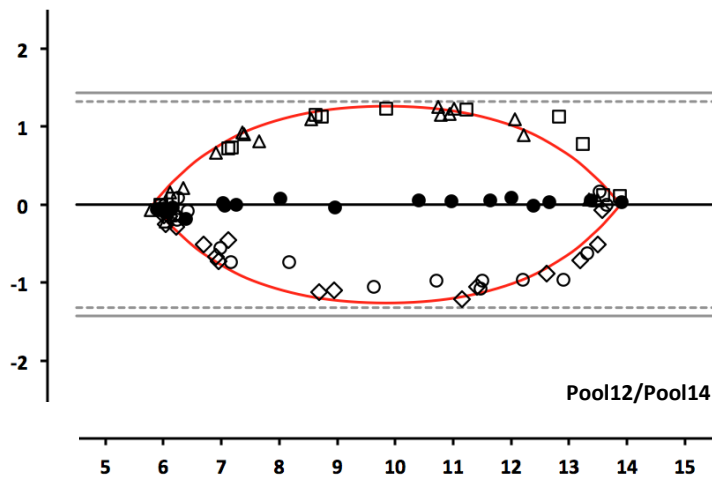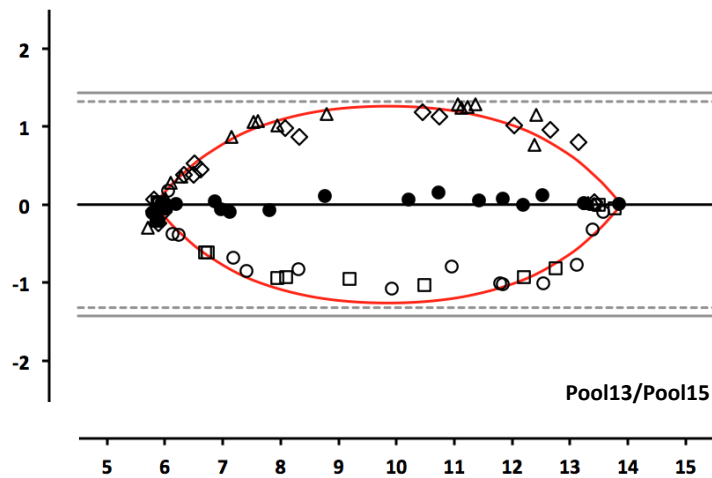

Average Log2 Signal

Supplement: Additional file 1: Figure S1. — Bland-Altman plot of each pair-wise pool comparison using the Illumina microarray platform. Symbols correspond to pools A-E (see Fig. 1). Filled circles = A, open circles = B, open diamonds = C, open triangles = D, and open squares = E. The red line corresponds to the ratio versus average intensity derived from the fitted Langmuir model. (PDF 249 kb) [file 12896_2016_281_MOESM1_ESM.pdf]

Log2 Ratio

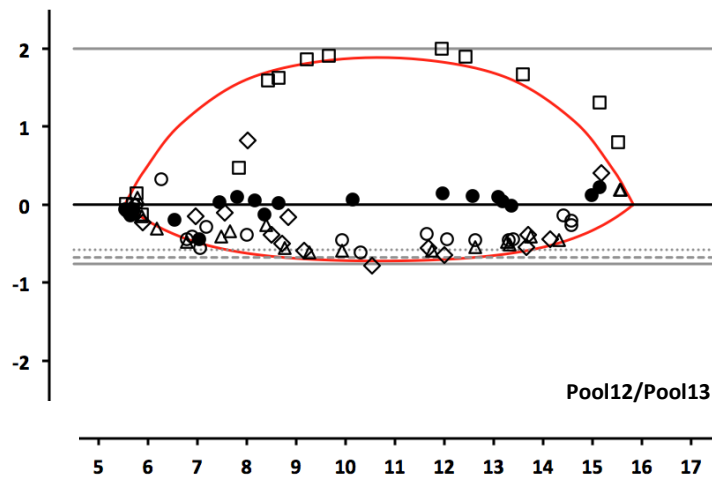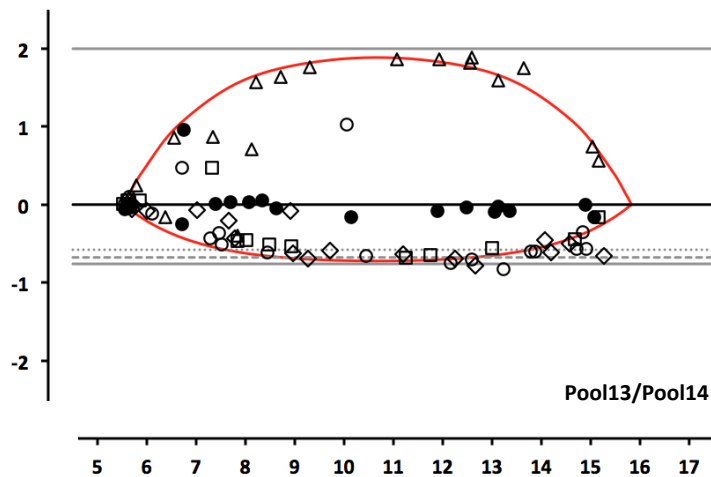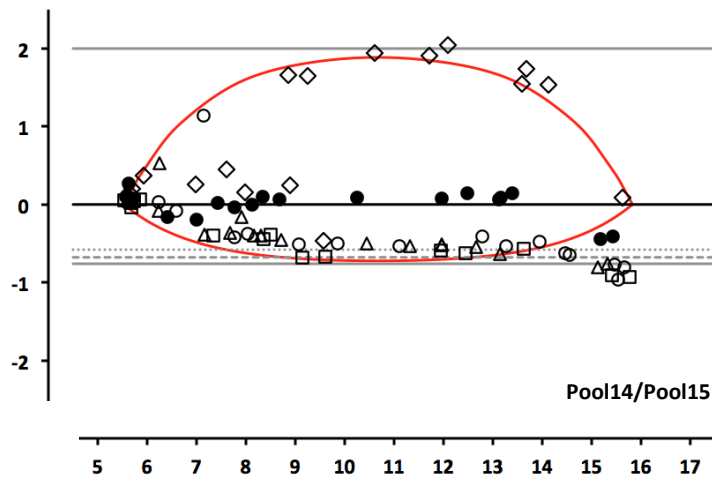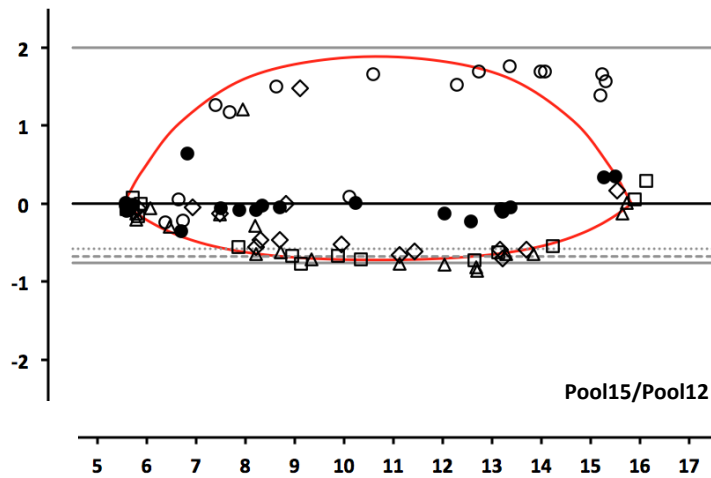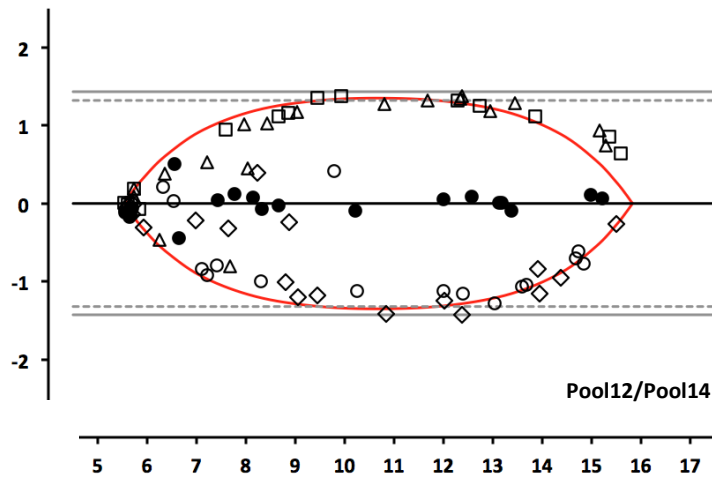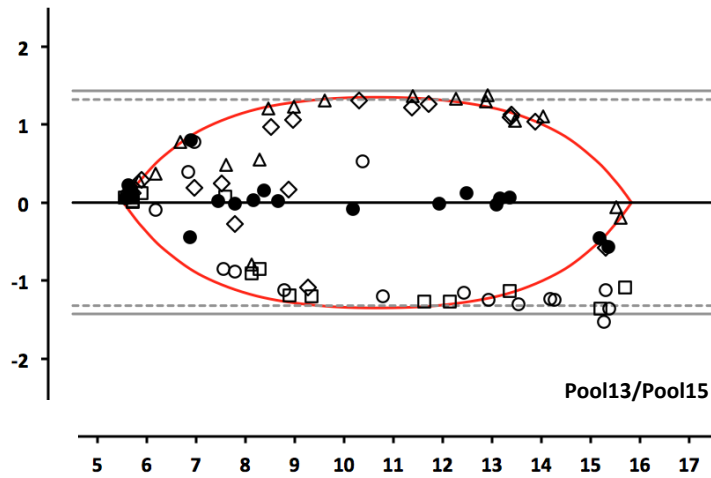

Average Log2 Signal

Supplement: Additional file 2: Figure S2. — Bland-Altman plot of each pair-wise pool comparison using the NIAID microarray platform. See Additional file 1: Figure S1 legend. (PDF 262 kb) [file 12896_2016_281_MOESM2_ESM.pdf]

Log2 Ratio

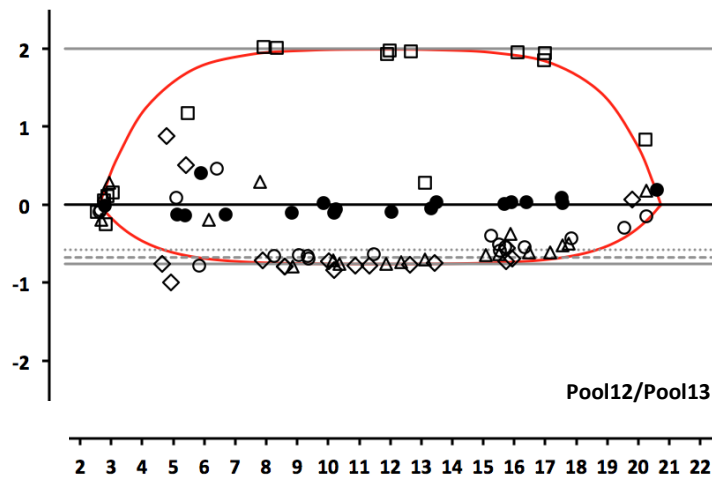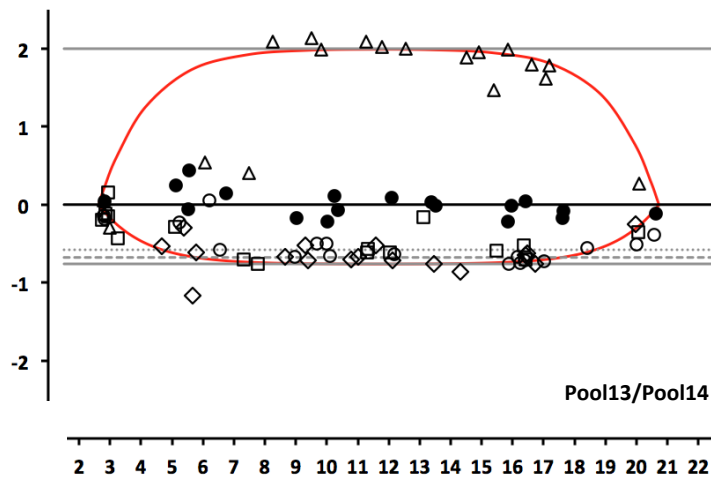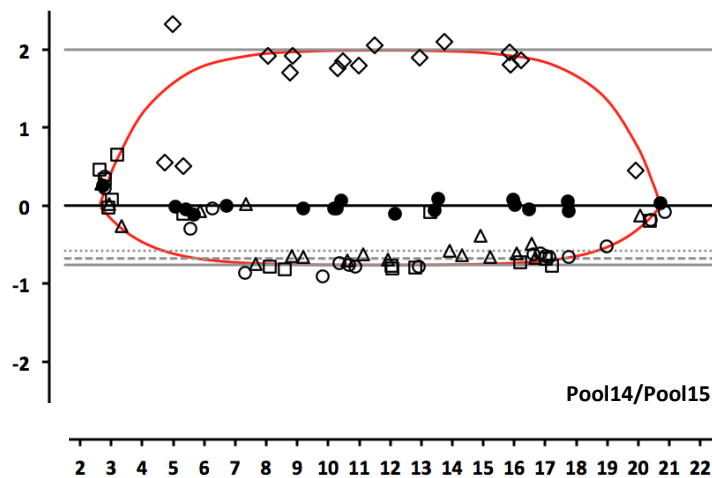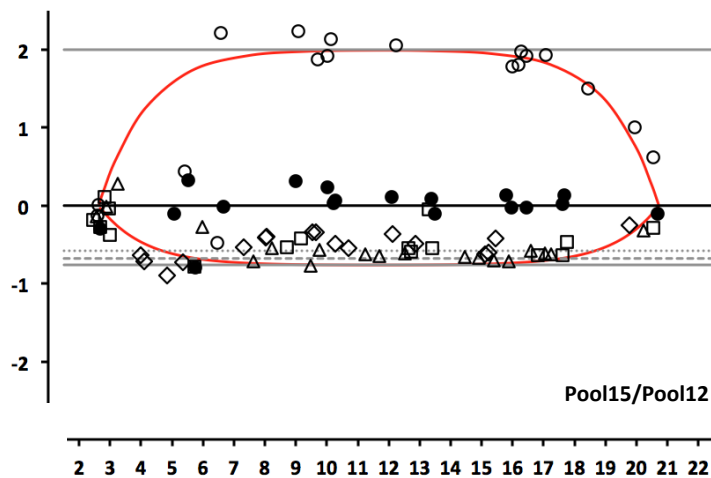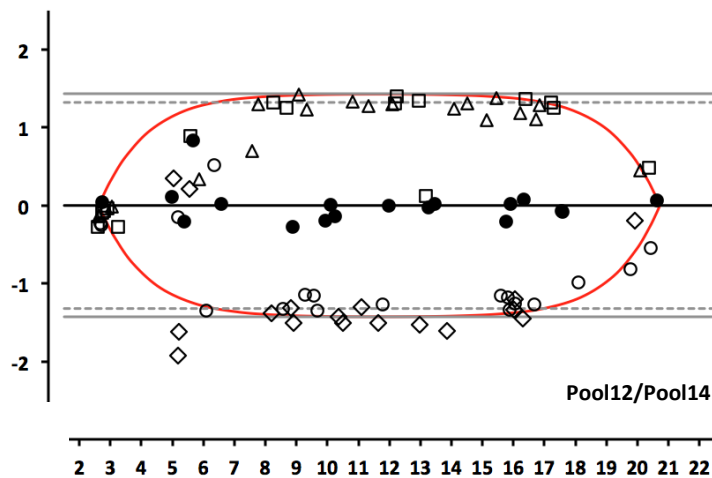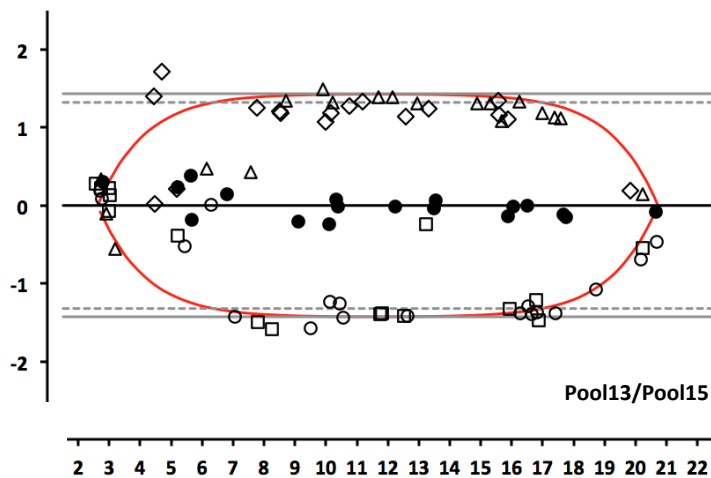

Average Log2 Signal

Supplement: Additional file 3: Figure S3. — Bland-Altman plot of each pair-wise pool comparison using the Agilent 1-color microarray platform. See Additional file 1: Figure S1 legend. (PDF 279 kb) [file 12896_2016_281_MOESM3_ESM.pdf]

Log2 Ratio

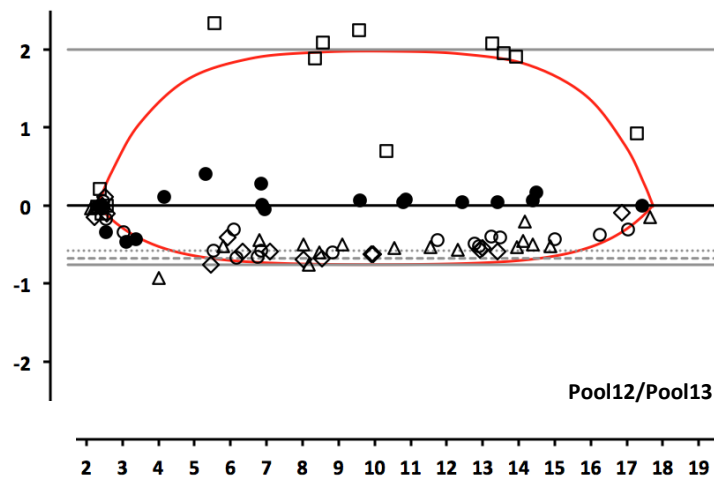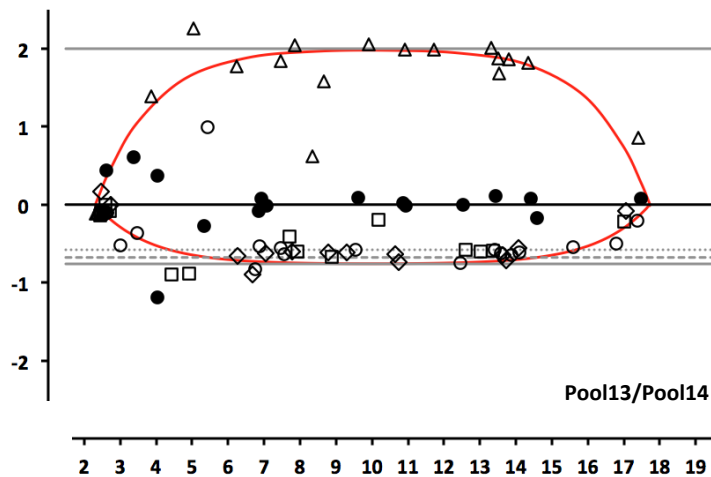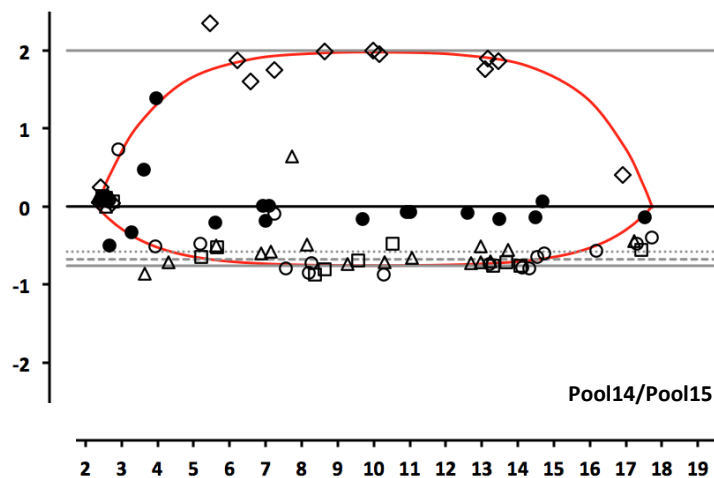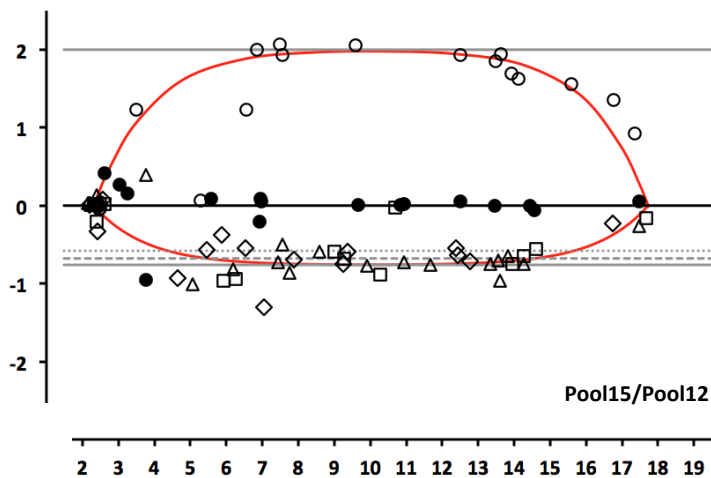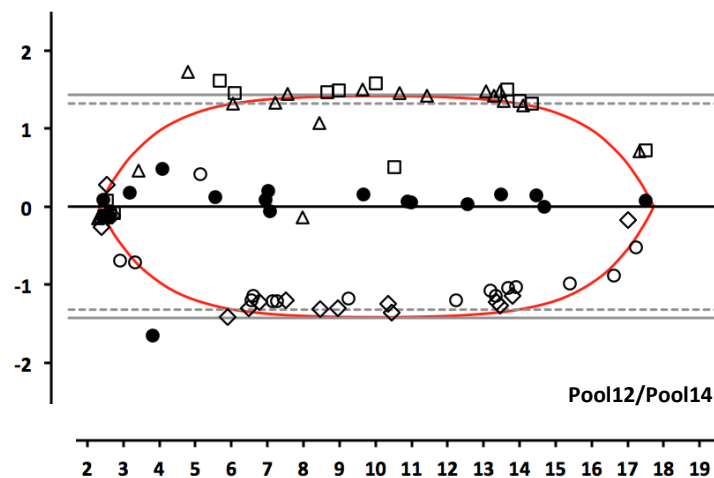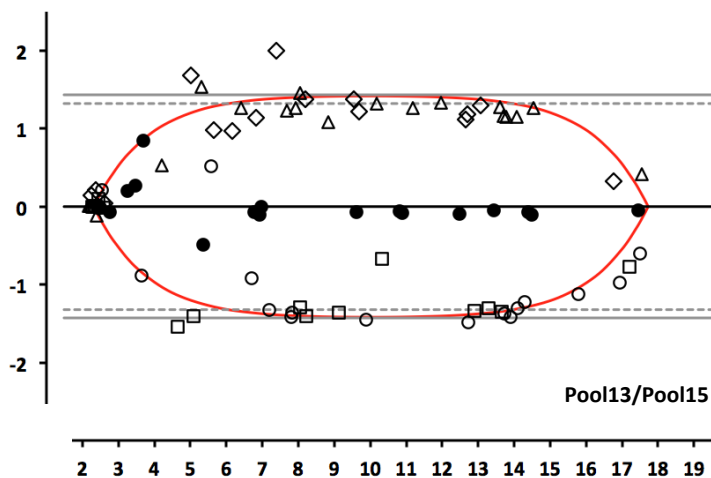

Average Log2 Signal

Supplement: Additional file 4: Figure S4. — Bland-Altman plot of each pair-wise pool comparison using the Agilent 2-color microarray platform. See Additional file 1: Figure S1 legend. (PDF 270 kb) [file 12896_2016_281_MOESM4_ESM.pdf]

Log2 Ratio

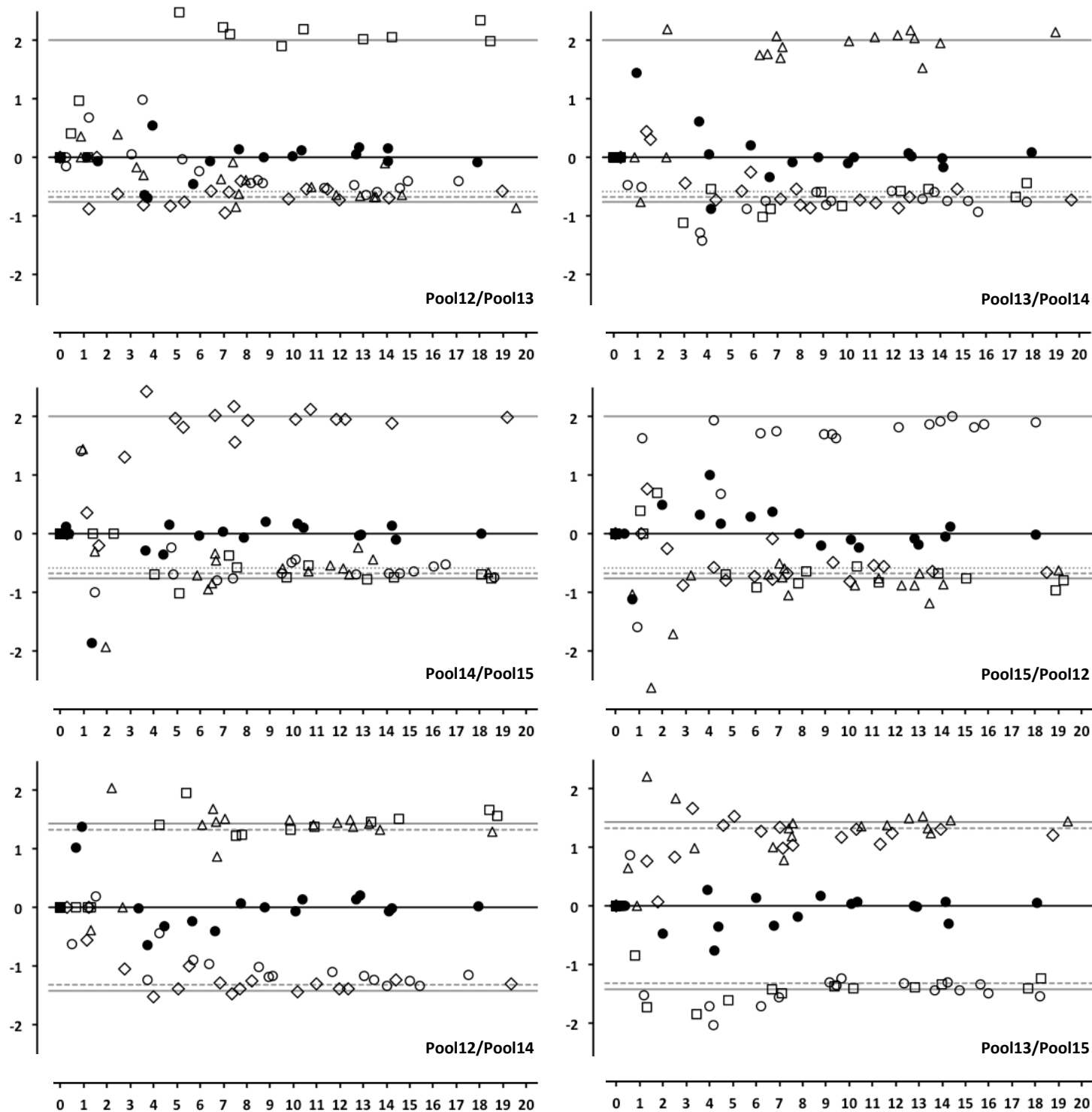

Average Log2 Signal

Supplement: Additional file 5: Figure S5. — Bland-Altman plot of each pair-wise pool comparison using the LifeTech NGS platform. Symbols correspond to subpools A-E (see Fig. 1). Filled circles = A, open circles = B, open diamonds = C, open triangles = D, and open squares = E. (PDF 186 kb) [file 12896_2016_281_MOESM5_ESM.pdf]

Log2 Ratio

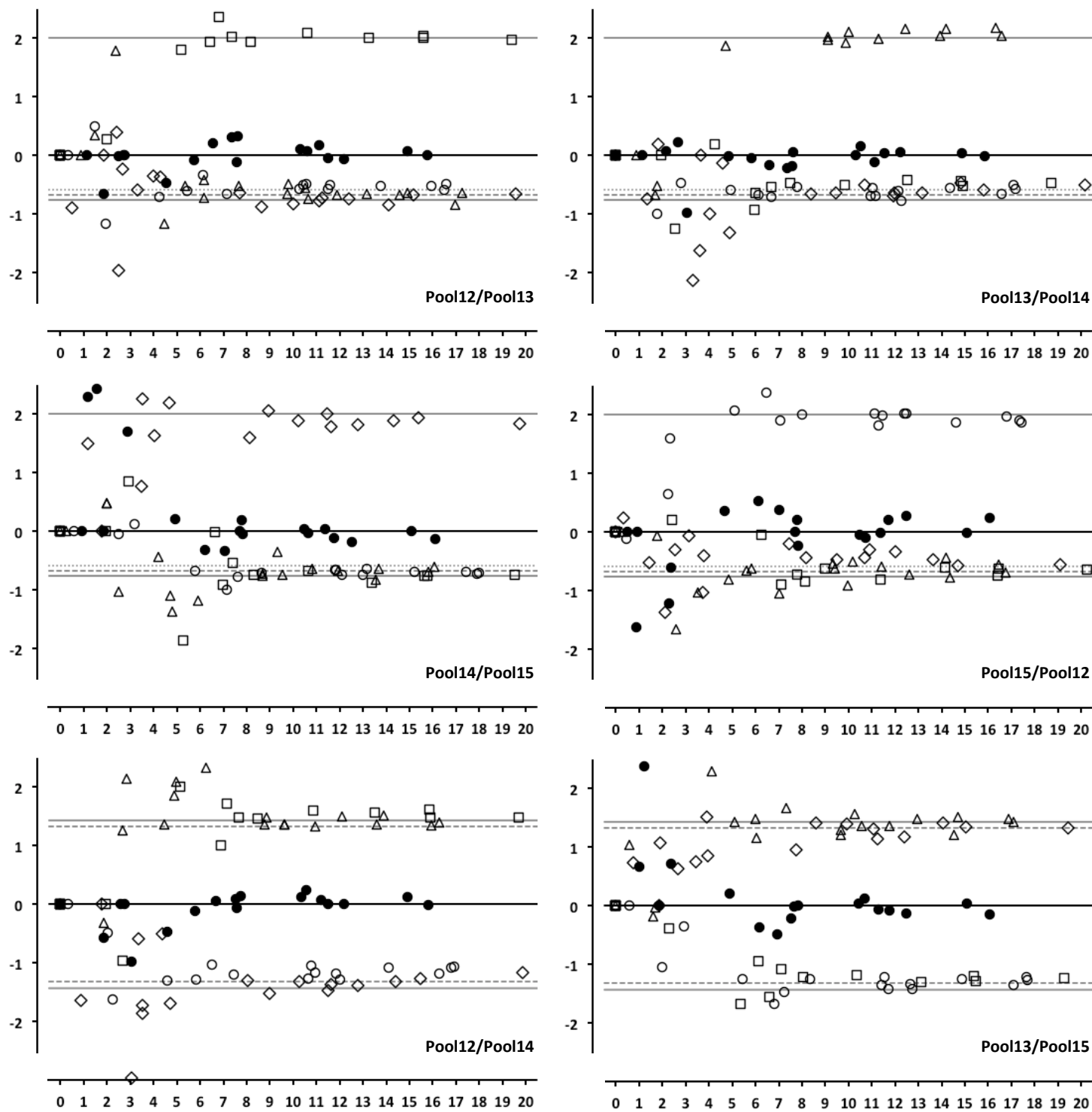

Average Log2 Signal

Supplement: Additional file 6: Figure S6. — Bland-Altman plot of each pair-wise pool comparison using the Illumina NGS platform. See Additional file 5: Figure S5 legend. (PDF 172 kb) [file 12896_2016_281_MOESM6_ESM.pdf]
